# Monocytes serve as Shiga toxin carriers during the development of hemolytic uremic syndrome

**DOI:** 10.1186/s11658-025-00689-8

**Published:** 2025-01-27

**Authors:** Xinlei Sun, Shuang Qu, Fenglian Zhou, Fujie Shi, Yunfei Wu, Lin Gu, Minghui Liu, Zhen Bian, Lei Shi, Zhihong Liu, Yuan Liu, Ke Zen

**Affiliations:** 1 State Key Laboratory of Pharmaceutical Biotechnology, Department of Gastroenterology, Drum Tower Hospital, Nanjing University Medical School, Nanjing University, Nanjing, 210093 Jiangsu China; 2 Geriatric Hospital of Nanjing Medical University, Nanjing, 210024 Jiangsu China; 3 School of Life Science and Technology, China Pharmaceutical University, 639 Longmian Avenue, Nanjing, 211198 Jiangsu China; 4 Jiangsu Provincial Central for Disease Prevention and Control, Nanjing, 210009 Jiangsu China; 5 National Clinical Research Center of Kidney Diseases, Jinling Hospital, Nanjing University School of Medicine, Nanjing, 210002 Jiangsu China; 6 Department of Emergency Medicine, Nanjing Drum Tower Hospital, Nanjing University Medical School, Nanjing, 210093 China

**Keywords:** Shiga toxin, Hemolytic uremic syndrome, Monocyte, Neutralizing antibody, Single-cell sequencing

## Abstract

Shiga toxin (Stx)-induced hemolytic uremic syndrome (HUS) poses a life-threatening complication for which a definitive treatment remains elusive. To exert its cytotoxic effect on renal cells, Stx must be delivered from the infected intestines to the kidney. However, the mechanism underlying Stx delivery remains unclear. Here we pinpoint monocytes as the primary carriers responsible for transporting Stx2 to the renal region. Through single-cell sequencing analysis of Stx2-B-bound peripheral white blood cells sorted by flow cytometry, we observe that nearly all monocytes exhibit strong Stx2-B binding, whereas less than 10% of neutrophils are associated with Stx2-B, albeit with a lower affinity. Further examination of the single-cell dataset and cell binding assays suggest that monocytes likely bind to Stx2-B through the Toll-like receptor 4. Remarkably, Stx-laden monocytes demonstrate their ability to transport Stx2 to human renal glomerular endothelial cells (HRGEC), subsequently inducing apoptosis in HRGEC. In a mouse model of Stx1/2-positive EDL933 infection-induced HUS, the presence of Stx2-positive monocytes in peripheral blood and infiltrated kidney tissues was observed. Finally, depleting monocytes through the usage of a CD14 neutralizing antibody or blocking monocyte chemotaxis via inhibition of CCL2 notably mitigates kidney injury and dysfunction caused by lipopolysaccharide (LPS)/Stx2 treatment. Our findings unveil the pivotal role of monocytes in Stx delivery during STEC infection and offer a promising therapeutic approach for Stx-induced HUS.

**Supplementary Information:**

The online version contains supplementary material available at 10.1186/s11658-025-00689-8.

## Introduction

Infection by intestinal Shiga toxin (Stx)-producing *Escherichia coli* (STEC) remains a serious public health problem worldwide. Although diarrhea is the major symptom, mortality from STEC infection is mainly caused by the extra-intestinal Stx-induced severe complication, termed hemolytic uremic syndrome (HUS), to which no specific treatment is available. STEC produces two main types of bipartite toxins, Stx1 and Stx2, which bind to glycolipid receptors (e.g., Gb3) highly expressed on target cells such as renal epithelial/endothelial cells [1, 2]. Following the endocytosis in target cells, Stx damages ribosomes and DNA, leading to cell apoptosis [3, 4]. The free toxin has been detected in the serum of HUS patients [5–8], although its role in HUS development has not been well characterized. It is generally believed that Stx is carried by certain circulating cells [9], yet the cell type that delivers Stx from the STEC-infected intestines to the kidney remains undefined.

During recent years, how Stx is transported from the infected intestines to distal organs has been extensively investigated [10–16]. Previous studies suggest neutrophils (PMNs) as a possible carrier for Stx in the peripheral circulation [10, 11]. Brigotti et al. [12] further identified Toll-like receptor 4 (TLR4) as a potential receptor of Stx in mediating the binding of PMNs with Stx. In line with this, a significant reduction in Stx transport to the Golgi by depleting TLR4 in colon carcinoma cells has been observed [16]. However, the reports about Stx–PMN binding are controversial. Multiple studies either failed to replicate Stx binding to human PMN or demonstrated that the binding was nonspecific or weak [13, 14]. An in vitro study by Winter et al. [15] showed that Stx did bind to leukocytes; however, they failed to identify which leukocyte bound to the toxin. Our previous study demonstrated that LPS-primed CD11b^+^ leukocytes served as an effective carrier of Stx2 in HUS development in mice [17]. Given that peripheral leukocytes, including PMNs, monocytes, and eosinophils, etc., all express CD11b, which leukocyte subset delivering Stx during HUS development is unclear. Employing non-human primate models of STEC infection, Stearns-Kurosawa et al. [18] found a marked infiltration of eosinophils in injured kidney tissues in the STEC-induced HUS, suggesting that eosinophils may play a role in carrying Stx to the kidney. Moreover, Stahl et al. [19] have revealed that platelets can bind toxins, undergo endocytosis, and release extracellular vesicles that facilitate toxin transfer. Further studies are required to characterize the role of platelets and their microvesicles in STEC-induced HUS development.

In this study, we utilized two distinct murine models to delve into the mechanisms behind the transport of Stx2 to the kidneys, where it triggers HUS. Specifically, we employed the LPS/Stx2-induced murine HUS model and the Stx1/2-positive EDL933 infection-induced murine HUS model. Through single-cell sequencing of Stx2-bound peripheral white blood cells and direct cell–toxin binding assays, we pinpointed circulating CD11b^+^CD14^+^ monocytes as highly effective carriers for Stx2. When these monocytes were briefly exposed to Stx2, they exhibited toxin binding capability, and transported the toxin to renal endothelial cells, leading to renal cell apoptosis. Employing the EDL933 infection-induced mouse HUS model, we observed a significant number of Stx2-positive monocytes in both peripheral blood and kidney tissues. Furthermore, we demonstrated that depleting peripheral monocytes through intravenous injection of a CD14 neutralizing antibody, or inhibiting monocyte chemokine CCL2 (MCP-1) by bindarit, significantly alleviated kidney injury and dysfunction in LPS/Stx2-treated mice. These findings shed light on the crucial role of monocytes in the delivery of Stx in the context of HUS associated with STEC infections, opening up potential therapeutic avenues for managing this serious condition.

## Materials and methods

### Cell culture

The use and handling of human blood samples in this study were approved by the Institutional Review Boards of the Chinese Pharmaceutical University (Nanjing, China), and written informed consent was obtained from each participant. Human peripheral blood immune cells were isolated from the whole blood of volunteers. Briefly, fresh heparinized blood from healthy human volunteers was centrifuged (200 ×*g*, 10 min) to separate the platelet-rich plasma and blood cells. After removing platelets, red blood cells were removed by NH_4_Cl lysis solution, and then the obtained cells were subjected to subsequent related experiments. THP-1 (human leukemia monocytic cell line), HK2 (human kidney proximal tubular cell line), HeLa (used to verify binding ability with Stx2-B-FITC and Stx2-CY5), and PANC-1 (served as a control with low expression of CD77 and TLR4) cell lines were obtained from the Shanghai Institute of Cell Biology (Shanghai, China). Human renal glomerular endothelial cells (HRGECs) were obtained from BeNa Culture Collection. The human podocyte cell line (HPC) was a gift from M. Saleem (Children’s Renal Unit, Bristol Royal Hospital for Children, University of Bristol, Bristol, United Kingdom). THP-1 cells were cultured in 1640 medium with 10% fetal bovine serum (FBS) and 0.1% β-mercaptoethanol. HeLa and PANC-1 cells were maintained in DMEM medium with 10% FBS. HK2 cells were cultured in DMEM/F12 medium with 10% FBS. HRGECs were grown in ECM medium (Sciencell) with 10% FBS. HPCs were cultured in a medium containing 10% FBS and insulin–transferrin–selenium (ITS-G, Gibco, cat. no. 41400-045, Canada) at 33 °C and subsequently differentiated at 37 °C over a period of 5 days.

### Stx2-B purification and FITC chemical conjugation

The gene encoding Stx2-B was cloned into the pET-21a plasmid using DNA from the STEC strain EDL933, and subsequently validated by DNA sequencing. The pET-21a plasmid, harboring the Stx2-B encoding sequence, was expressed in *E. coli* BL21 (DE3) cells. The bacterial culture was cultivated overnight at 37 °C in LB media supplemented with 50 μg/ml kanamycin. Upon reaching an OD600 of 0.8, cells were induced by adding 0.5 mM isopropyl β-d-thiogalactoside (IPTG) and further cultured for an additional 20 h at 16 °C. After incubation, bacteria containing the recombinant Stx2-B protein were harvested via centrifugation and sonicated in a cold lysis buffer (50 mM Tris–HCl, pH 8.0, 500 mM NaCl, 5 mM imidazole, 1 mM TCEP, and 1% complete EDTA-free protease inhibitor tablets). Subsequently, the lysate was centrifuged at 15,000 ×*g* for 45 min at 4 °C. The supernatant was applied to a chromatography column packed with Ni Sepharose 6 Fast Flow beads (GE Life Sciences) in working buffer (50 mM Tris–HCl, pH 8.0, 500 mM NaCl, 5 mM imidazole, and 1 mM tris[2-carboxyethyl]phosphine [TCEP]). Stx2-B protein was eluted using a working buffer containing 250 mM imidazole. For further purification, the eluted protein underwent gel filtration using a Superdex 200 column (GE Life Sciences) in FPLC buffer (50 mM Tris–HCl, pH 8.0, 150 mM NaCl, 1 mM TCEP). The fractions containing the Stx2-B protein were combined and validated using Coomassie blue staining. Subsequently, it was chemically conjugated with fluorescein isothiocyanate (FITC) using the LinKine FITC Labeling Kit (Abbkine, KTL0210). Briefly, purified Stx2-B (0.1 mg) was incubated with 5µL FITC labeling solution and 2.5µL activated solution for 1 h (25 ℃). After that, Stx2-B-FITC is obtained by column centrifugation (12,000*g*, 10 min) to remove the excess uncoupled FITC. The effectiveness of Stx2-B-FITC was verified through immunofluorescence by incubating with HeLa cells [20] for 30 min at 37 °C.

### Single-cell preparation and single-cell RNA-sequencing (scRNA-seq)

Human peripheral blood immune cells were incubated with Stx2-B-FITC for 30 min at 37 °C. Stx2-B-positive cells were isolated via flow cytometry sorting (BD FACS Aria SORP). The resulting cells underwent manual counting three times with trypan blue, ensuring that over 95% of the cells remained viable after each centrifugation. The cells were then resuspended at a concentration of ≥ 2 × 10^5^/ml. Single cells were processed using a Chromium Controller (10x Genomics) following the manufacturer’s protocol [21]. Subsequently, these single cells were run on a 10x Chromium system and underwent library preparation by LC Sciences, following the recommended protocol for the Chromium Single Cell 30 Reagent Kit (v2 Chemistry), with sequencing performed using the HiSeq4000 for Illumina. Post-processing and quality control were carried out using the 10x Cell Ranger package (v1.2.0; 10x Genomics). All reads were aligned to the GRCh38 reference assembly (v96). The initial assessment using the 10x Cell Ranger for the Stx2-B-positive sample reported 9410 cell barcodes with a median of 2437 genes per cell sequenced, achieving 97.0% sequencing saturation with a mean of 45,584 reads per cell. Bioinformatic analysis of the single-cell RNA sequencing data with 8681 quality-filtered cells was conducted using the R package Seurat (version 3.0) with default parameters by LC-bio (Hangzhou, China). Low-quality cells, defined by criteria such as minimum expression cells < 3, gene numbers < 200, and mitochondrial genes > 10%, were filtered out. Clustering analysis was performed with standard Seurat package procedures, using a resolution of 1.2. The identified clusters were visualized using the uniform manifold approximation and projection (UMAP) of the principal components in Seurat. For the analysis of *A4GALT* and *TLR4* gene expression in different cells and the alteration of the *Ccl* levels in mouse kidneys treated with LPS and Stx2 for 6 h, deposited datasets from the Gene Expression Omnibus (GEO) repository (accession number GSE202109 [22] and GSE180476 [23]) were analyzed using DotPlot or visualized in a heatmap using the ggplot2 package.

### Stx2 holotoxin-CY5 chemical conjugation

The holotoxin of Stx2 (Toxin Technology Inc, 11420V2) was chemically conjugated with Cy5 (MCE, HY-D0819A). Briefly, 10 μl reaction buffer (1 M sodium carbonate solution, ~ pH 9.0) was mixed with 90 μl Stx2 protein solution in phosphate buffered saline (PBS) at pH 7.2–7.4, achieving a final concentration of 2 mg/ml at ~ pH 8.5. The mixture was then mixed with 10 μl of 1.2 mg/ml Cy5 mono-NHS-ester in 10% DMSO and incubated for 2 h on ice. The Stx2-CY5 conjugate was then purified using Zeba™ Dye and Biotin Removal Spin Columns (Thermo Scientific, A44296S). The effectiveness of Stx2-CY5 on HeLa cell binding was verified by flow cytometry.

### Toxin–cell binding affinity assay

To assess the binding of Stx2 with monocytes, white blood cells (1 × 10^6^) were isolated from human peripheral blood and incubated with Stx2-B-FITC (100 ng/ml) or Stx2-CY5 (2 ng/ml) in 0.5 ml PBS for 30 min or indicated time points (0, 0.5, 1, 3, and 6 h, respectively). After labeling, the cells were washed and labeled with fluorescent anti-human CD14 (BioLegend) and anti-human CD11b (BioLegend) antibodies, followed by flow cytometry (Bekman, USA) or immunofluorescence analysis (Leica, Germany). To assess the binding affinity of different cells to Stx2, THP-1 cells and HRGECs were incubated with different concentration of either Stx2-B-FITC or Stx2-CY5 for 30 min. The cells were washed and analyzed by flow cytometry.

### Toxin delivery by monocytes to renal endothelial cells

To assess the delivery of toxin by monocytes and its impact on renal endothelial cells, we investigated renal cell death as an indicator. In this experimental setup, THP-1 cells labeled with carboxyfluoroscein succinimidyl ester (CFSE) (Invitrogen, C34554) were incubated with Stx2 at a concentration of 2 ng/ml. Subsequently, these cells were thoroughly washed with PBS and co-cultured with HRGECs. After 24 h, the THP-1 cells were removed, and HRGEC apoptosis was assessed using the apoptosis detection kit from Keygen (KGA1030). For the cytotoxicity assay, 2 × 10^4^ HRGECs were seeded in 96-well plates. The Stx2-bound THP-1 cells were co-cultured with HRGECs for 24 h. Following an extensive wash with PBS to eliminate THP-1 cells, 10 μl of CCK-8 reagent (CK04-500, Dojindo, Japan) was added to measure the cell cytotoxicity index in accordance with the manufacturer’s protocol. The absorbance value was then determined at a wavelength of 450 nm using a SpectraMax instrument (Molecular Devices, USA).

### Toxin–extracellular vesicles (EVs) binding assay

To assay the potential of EVs released from monocytes or neutrophils in delivery of Stx2, we isolated human monocytes (CD11b^+^CD14^+^) or neutrophils (CD11b^+^CD14^−^) and then treated the cells with Stx2-B-FITC (100 ng/ml) or Stx2 (2 ng/ml) at 37 °C. The EVs released by these cells were collected at different time points (0, 0.5, 1, 3, and 6 h) from the supernatant using magnetic wheat germ agglutinin (WGA)-beads (~ 2 μm size) for further purification [24]. The WGA-beads were coupled using NHS magnetic beads (Solarbio, M2450) and wheat germ agglutinin (Invitrogen, W7024) according to the manufacturer’s instructions. The EVs collected at different time points were then incubated with HRGECs. After 24 h incubation, HRGEC apoptosis was detected using the Keygen apoptosis detection kit (KGA1030).

### Docking of Stx2-B and TLR4

For protein docking, we obtained the extracellular protein sequence of TLR4 (O00206) and the protein sequence of Stx2-B (P09386) from the Swiss-Prot database. We then built the protein docking model using the AlphaFold Server. The results were processed and visualized using PyMOL.

### TLR4 neutralization assay

Anti-human CD77/Gb-3 (BioLegend, 357103) and CD284/TLR4 (BioLegend, 312815) were used to detect Gb-3 and TLR4 in various cells, respectively. To explore the impact of a TLR4 neutralizing antibody (BioLegend, 312813) on the binding between Stx2-B and THP-1 cells, an initial treatment with dye Dil (Beyotime, C1036) was administered. Subsequently, the cells were incubated with Stx2-B-FITC along with the TLR4 neutralizing antibody (10 μg/ml) or the corresponding Mouse IgG2a for a duration of 30 min, followed by immunofluorescence imaging (Leica, Germany).

### Mice

The *Escherichia coli* (*E. coli*) O157:H7 (EDL933) strain was obtained from Jiangsu Provincial Center for Disease Control and Prevention. Male C57BL/6 mice (6 weeks old) were obtained from the specific-pathogen-free (SPF) facilities of the Model Animal Research Center of Nanjing University (Nanjing, China), and CD-1 mice (4–6 weeks old) were obtained from the specific-pathogen-free (SPF) facilities of Vital River (Beijing, China). The EDL933-infected mouse model [25] was established for this study. Briefly, the male CD-1 mice were provided 5 g/l streptomycin sulfate (BBI, 3810-74-0) in their drinking water and an overnight fast, then inoculation with 10^10^ colony formuing units (CFU) of str-resistant EDL933 in 20% sucrose (w/v). During the infection model, the mouse status was observed and recorded. Mice were euthanized after 6 days, and the tissue was collected and analyzed. In the EDL933-infected mice model, white blood cells (WBCs) isolated from EDL933-infected mice and uninfected mice were incubated with anti-mouse CD14 (BioLegend, 150105) for 30 min, followed by flow cytometry analysis. The LPS/Stx2-induced mouse HUS model [17] was used in this study. Male C57BL/6 mice were first administered intraperitoneally with 300 μg/kg LPS (*E. coli* O111:B4 strain, Sigma-Aldrich, L2630). After 24 h, mice were injected with 500 ng/kg Stx2 intraperitoneally (i.p.) (Toxin Technology Inc, 11420V2) for three times at every 24 h interval, then accompanied by i.p. injection of 50 μg CD14 neutralizing antibody every day for 3 days (BioLegend, 150103) or administration with bindarit (100 mg/kg/day, MCE, HY-B0498) through gavage. Mice were monitored daily, and the tissues were collected and analyzed after being sacrificed at 72 h. Plasma creatinine levels were determined using the Creatinine Assay Kit (Sigma, MAK080).

### Immunofluorescence

For immunofluorescence of renal, mice tissues were frozen in optimal cutting temperature (OCT) compound for sectioning. Renal sections of 5 μm were fixed in 4% paraformaldehyde and blocked in 10% donkey serum for 30 min. We used anti-Stx2 (Thermo Fisher, MA5-33371), anti-CD14 (Abcam, ab221678), anti-CD11b (R&D, MAB1124), anti-F4/80 (R&D, MAB5580), anti-CD3 (Abcam, ab5690), and anti-CD8 (R&D, MAB116) as primary antibodies. After incubating with antibodies overnight at 4 °C, tissue sections were incubated with anti-Mouse-Alexa Fluor^TM^ 488 (Thermo Fisher, A-21202) or anti-Mouse-Alexa Fluor^TM^ 594 for Stx2 (Thermo Fisher, A-21203), anti-Rabbit-Alexa Fluor^TM^ 488 (Thermo Fisher, A-21206) for CD14 and CD3, and anti-Rat-Alexa Fluor^TM^ 594 (Thermo Fisher, A-21209) for CD11b, F4/80, and CD8, respectively. The secondary antibody incubation was carried out at room temperature for 1 h. Notably, kidney sections incubated with a secondary antibody served as the negative control (Fig. S5). After washing three times, tissue sections were stained briefly with DAPI (Santa Cruz) and then mounted by AntiFade mountant (Thermo Fisher). A confocal microscopy study was performed on the confocal microscope (Leica, Germany).

### Western blot

Protein extraction and western blotting were performed as described previously [26]. Protein levels were normalized to β-actin and analyzed by ImageJ software. The antibodies used in this study are: anti-Bax (CST, no. 41162), anti-Cleaved Caspase-3 (CST, no. 9664), and anti-β-actin (CST, no. 4970). Uncropped data of western blots are presented in the Supplementary File.

### Histopathological analysis

Renal tissues were fixed with 4% paraformaldehyde and then infiltrated with and embedded in paraffin after dehydration and clearing. After deparaffination and rehydration, the sections were stained separately with PAS and H&E staining. Photomicrographs of the sections were obtained with a light microscope (Olympus, Japan). The extent of tubular damage was determined by assessing renal tubular injuries such as cell atrophy and dilation.

### Statistical analysis

The data in this study were derived from a minimum of three independent experiments and are expressed as the mean ± standard error of the mean (SEM). Survival curves were subjected to analysis using the Log-rank (Mantel-Cox) test. Statistical significance was determined with a threshold of *P* < 0.05, employing either one-way analysis of variance (ANOVA) (followed by Tukey’s multiple comparisons test), two-way ANOVA (followed by Bonferroni’s multiple comparisons test), or a two-tailed *t*-test, as appropriate for the specific analysis. All the schematic diagrams were created with BioRender software.

## Results

### Identification of peripheral monocytes as Stx2 carriers

**Fig. 1:**
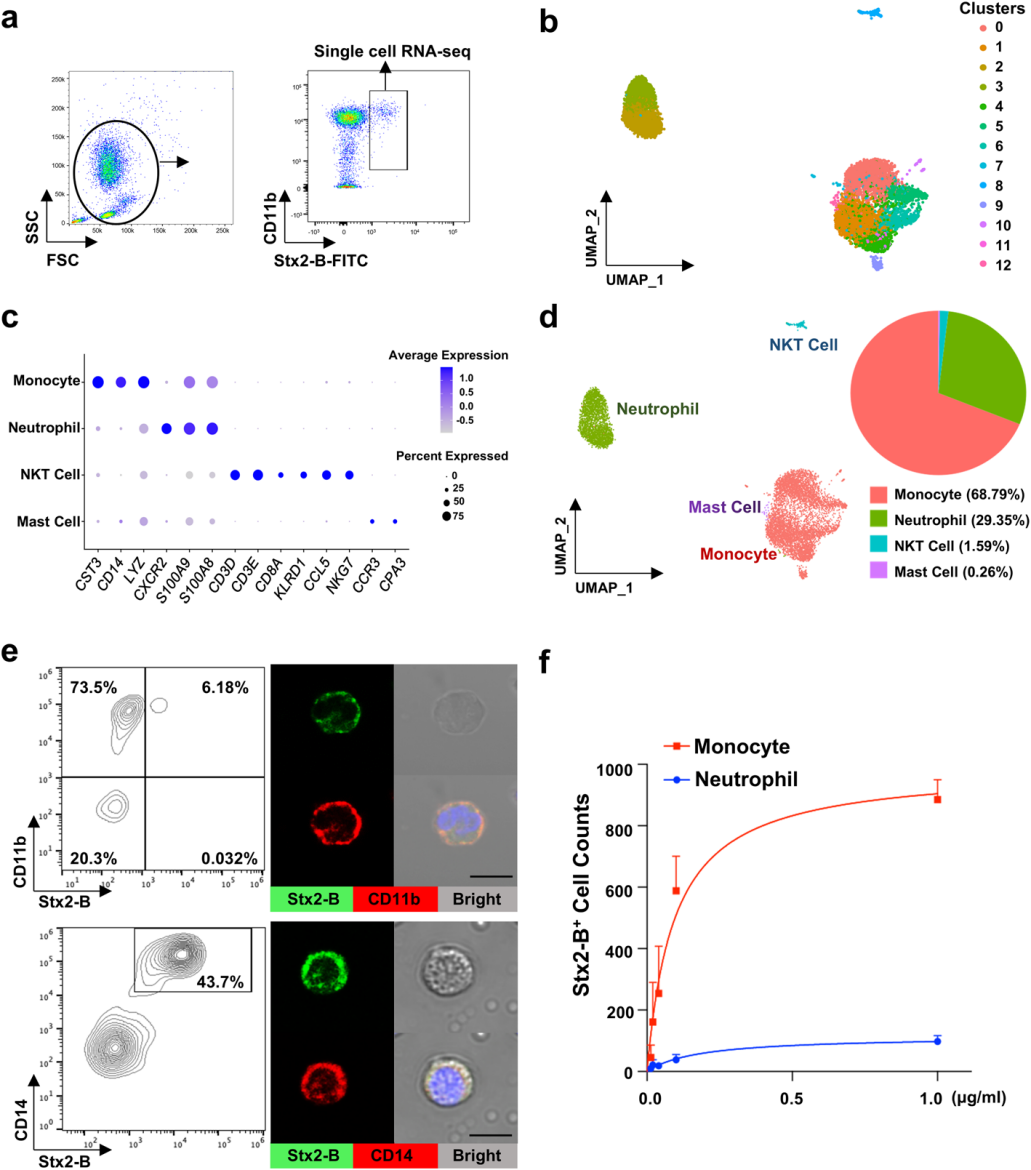
Identification of peripheral monocytes as Stx2 carriers by single-cell sequencing and flow cytometry. **a** Schematic of sorting Stx2-B-binding cells for single-cell sequencing analysis. **b** Major clusters of immune cells were seen in UMAP (left panel) and non-supervised clustering of 8681 quality-filtered, single-cell transcriptomes collected from Stx2-B-binding cells (right panel). **c** Major clusters of immune cells were seen in UMAP (left panel) and distribution of Stx2-B-positive immune cells (right panel). **d** Percentage of monocytes and neutrophils that bind to Stx2-B, analyzed by single cell sequencing. **e** Upper panel; partial CD11b^+^ cell binding to Stx2-B, detected by flow cytometry (left) and immunofluorescence label (right). Lower panel; almost total CD14^+^ monocyte binding to Stx2-B, detected by flow cytometry (left) and immunofluorescence label (right). The DAPI staining highlights cell nuclei (blue). Scale bar, 10 μm. **f** Binding efficiency of Stx2-B to neutrophils and monocytes detected by flow cytometry. Human peripheral blood immune cells were obtained from two individuals. Data are presented as mean ± SD

To identify the potential carrier for Shiga toxin in STEC-induced HUS, we first purified the toxin Stx2 B subunit (Stx2-B) from the STEC strain EDL933 [27] (Supplementary Fig. S1a). Stx2-B-positive HeLa cells were detected by immunofluorescence after incubating the cells with FITC-conjugated Stx2-B (100 ng/ml) for 30 min. It was observed that the toxin not only bound to the cell surface but also entered the cells following incubation at 37 °C (Supplementary Fig. S1b). Single-cell sequencing analysis (scRNA-seq) was used to identify the cell population in the human peripheral blood that could serve as a Stx2-B carrier candidate . Briefly, peripheral white blood cells from healthy donor were incubated with FITC-conjugated Stx2-B for 30 min at room temperature, the Stx2-B-positive cells were then sorted out by flow cytometry and subjected to single-cell sequencing (Fig. 1a). Major clusters of the Stx2-B-positive cells were shown in UMAP (uniform manifold approximation and projection) (Fig. 1b). The analysis of 8681 quality-filtered, single-cell transcriptomes (Fig. 1c) suggested that the Stx2-B-positive cells were mainly monocytes. In fact, monocytes occupied nearly 70% of all Stx2-B-bound cells in peripheral blood (Fig. 1d), while less than 30% of Stx2-B-bound cells were neutrophils. Given that neutrophils significantly outnumber monocytes in the peripheral blood, this suggests that monocytes have a higher affinity to Stx2-B compared with neutrophils. Given that this toxin can be cleaved [7], the relatively low binding of the toxin to neutrophils could be due to the cleavage of toxin.

We next examined the binding of Stx2-B with monocytes using flow cytometry and immunofluorescence labeling. As shown in Fig. 1e, only a small fraction of CD11b^+^ cells bind to Stx2-B, whereas CD14^+^ monocytes were nearly all bound to Stx2-B. As CD11b is expressed in both neutrophils and monocytes, this observation is in line with the notion that most monocytes and a small fraction of neutrophils can bind to Stx2. The cell–toxin binding assay further showed that monocytes, but not neutrophils, strongly bound to Stx2-B, and the monocyte–Stx2-B binding was dose-dependent (Fig. 1f). Expanding this analysis, we conducted experiments using the CY5-labeled Stx2 holotoxin. We verified the binding of Stx2-CY5 to HeLa cells via flow cytometry (Supplementary Fig. S2a). Consistent with the Stx2-B results, Stx2-CY5 strongly bound to CD14^+^ cells (Supplementary Fig. S2b).

### Monocytes deliver Stx2 to renal endothelial cells to induce cell apoptosis

**Fig. 2:**
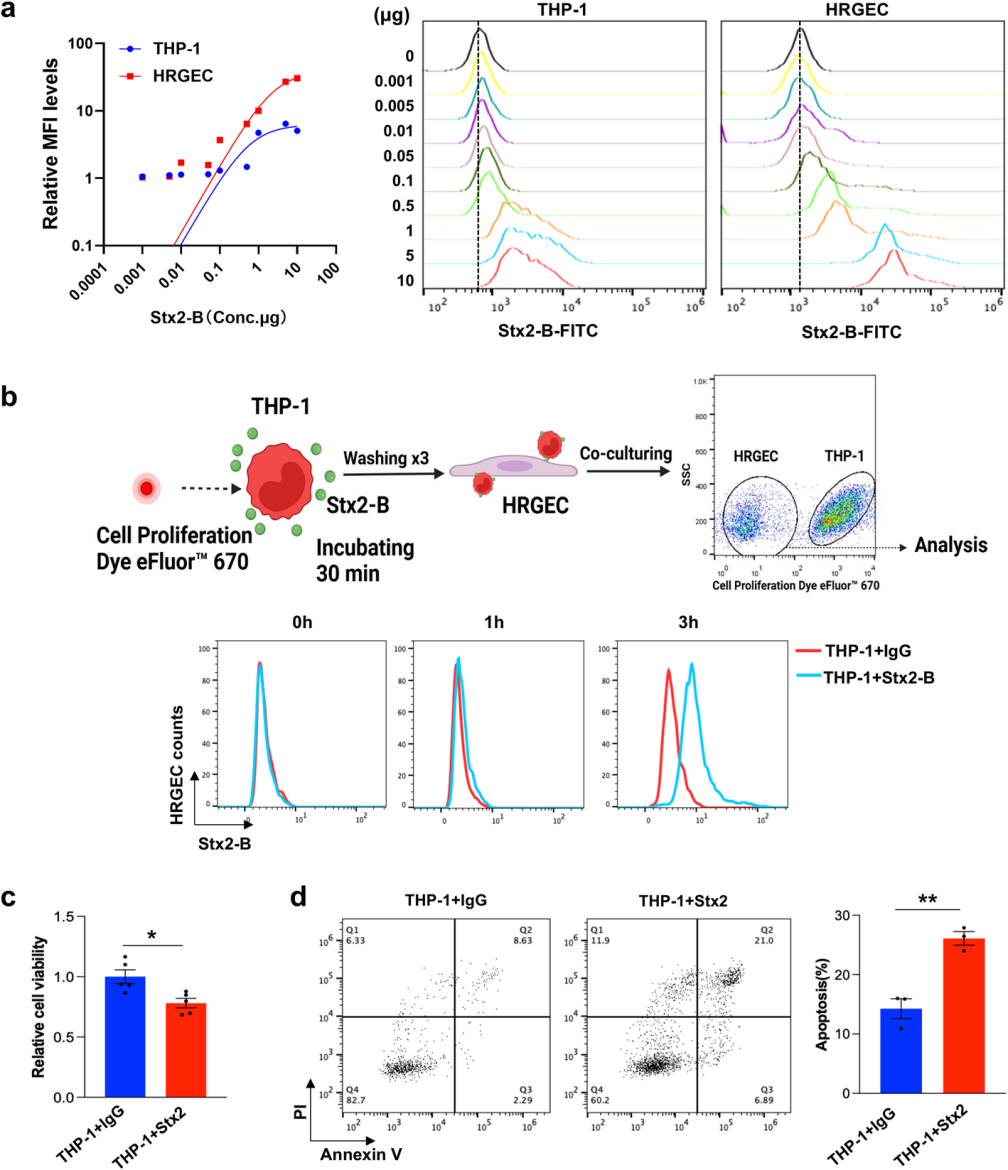
Monocytes deliver Stx2 to HRGECs to induce HRGEC apoptosis. **a** Binding capacity of Stx2-B to THP-1 monocytes and HRGECs. **b** Top; schematic diagram of THP-1 monocytes delivering Stx2-B to HRGECs. Bottom; level of Stx2-B-FITC on HRGEC surface following incubation with toxin-bearing THP-1 monocytes for 0, 1, and 3 h, respectively. **c** HRGEC viability was determined using a CCK-8 assay, after 24 h treatment with or without Stx2-bearing THP-1 monocytes. **d** HRGEC apoptosis after 24 h treatment with or without Stx2-bearing THP-1 monocytes. Statistical differences between groups in (**c**) and (**d**) were assessed by two-tailed unpaired *t*-tests. Data are presented as mean ± SEM. *P < 0.05. **P < 0.01

To determine whether monocytes can deliver Stx2 to renal endothelial cells, we compared the binding affinity of human monocytes (THP-1) and renal endothelial cells (HRGECs) with Stx2. As shown in Fig. 2a and Supplementary Fig. S2c, HRGECs displayed a significantly higher affinity to Stx2 than THP-1 monocytes, supporting the notion that THP-1-bearing toxin can be transferred to HRGECs owing to the difference in binding affinity. As shown in Fig. 2b, Stx2-B-FITC was clearly directly transferred from THP-1 monocytes to HRGECs after a 3 h incubation. Cell viability assays further indicated that incubation with Stx2-bearing THP-1 markedly reduced the viability of HRGECs (Fig. 2c). In line with this, cell apoptosis assays showed that incubation with Stx2-bearing THP-1 significantly induced HRGEC apoptosis (Fig. 2d). These results collectively suggest that monocytes can deliver Stx2 to renal endothelial cells to induce cell apoptosis in vitro.

Recent studies reported that Stx2 could enter the kidneys via cell-derived microvesicles (particulate Stx), leading to HUS-related diseases [5, 19]. To investigate whether monocytes transport Stx2 through releasing extracellular vesicles (EVs), we treated human peripheral monocytes, as well as PMNs, with Stx2-B-FITC for 0, 0.5, 1, 3, and 6 h, and then isolated the EVs from supernatant using magnetic WGA-beads (Supplementary Fig. S3a). Flow cytometry did not detect Stx2-B-FITC on EVs released by monocytes and PMNs at 0, 0.5, 1, and 3 h post-incubation (Supplementary Fig. S3b). Even after 6 h, EVs released by monocytes displayed a low level of Stx2-B-FITC, indicating that monocyte-released EVs are unlikely to significantly contribute to Stx2 delivery. To serve as a control, CD47 level on EVs was detected following incubation with Stx2 (Supplementary Fig. S3c). Moreover, we compared the level of Stx2-B-FITC on the surface of THP-1 and the EVs released by THP-1 following incubation with Stx2-B-FITC. THP-1 monocytes bound to Stx2-B-FITC in a time-dependent manner, whereas the EVs released by THP-1 monocytes displayed a weak association with Stx2-B-FITC even after a 6 h incubation (Supplementary Fig. S3d). In line with this, incubation with the EVs released by Stx2-treated monocytes (Supplementary Fig. S3e) or THP-1 (Supplementary Fig. S3f) did not induce significant apoptosis of HRGECs.

### The binding of Stx2 with monocytes is likely mediated by TLR4

**Fig. 3:**
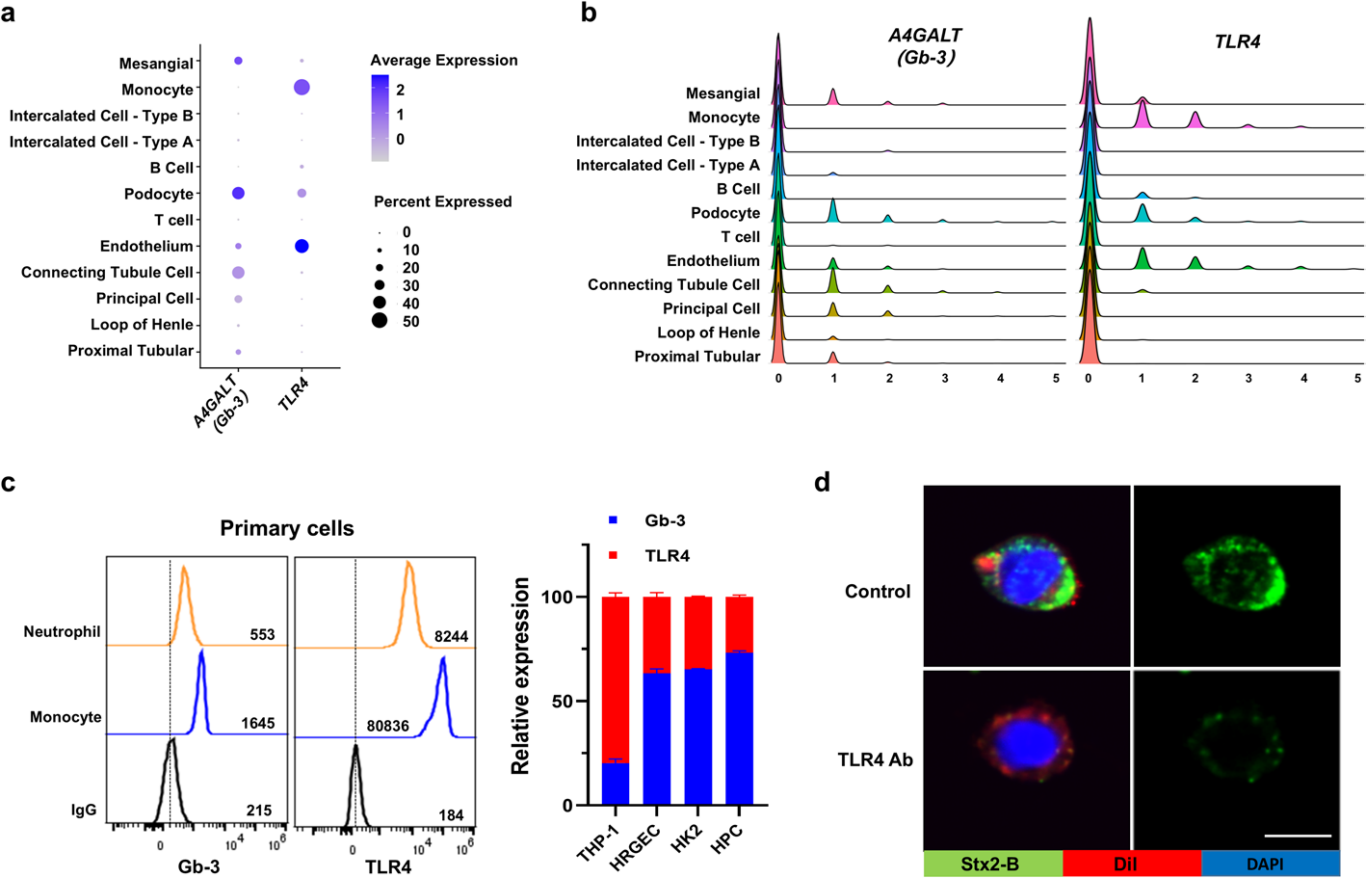
Expression of the Stx2 receptor in different cells in the renal microenvironment. **a** The expression level of *A4GALT* (*Gb3*) and *TLR4* in different types of cells from total human kidney homogenate using the DotPlot function. **b** The expression level of *A4GALT* (*Gb3*) and *TLR4* in different types of cells from total human kidney homogenate in a ridge plot. **c** Detection of CD77 (GB-3) and TLR4 expression in human peripheral monocytes and neutrophils (left) and various cell lines (right) by flow cytometry. **d** Immunofluorescence detection of a TLR4 neutralizing antibody interfering with the binding of Stx2-B to THP-1 cells. The DAPI staining was used to highlight the cell nuclei (blue), while the Dil staining was employed to visualize the cell membranes (red). Scale bar, 10 μm

It has been known that glycoprotein CD77 (Gb-3) serves as a Stx receptor on renal endothelial cells [28]. However, given that monocytes and HRGECs display different binding affinity to Stx2, we postulated that monocytes and HRGECs express different receptors for Stx2. To validate this hypothesis, we analyzed CD77 and TLR4, two receptors that have been reported to be responsible for Stx2 binding [12, 29], using single-cell RNA-sequences of a human kidney cell mixture (CEO dataset, GSE202109) [22]. In this study, the level of *A4GALT * (*Gb3*), a synthetase for glycoprotein CD77, was analyzed to reflect the differential expression of CD77. As shown in Fig. 3a, b, *TLR4* and *A4GALT * (*Gb3*) displayed different expression patterns, in which *TLR4* was significantly expressed in monocytes as well as endothelial cells, whereas *A4GALT * (*Gb3*) was mainly expressed in renal podocytes, tubular cells, and mesangial cells, etc. Flow cytometry analysis showed that monocytes and neutrophils strongly expressed TLR4 but not CD77 (Fig. 3c, left). Further experiments showed that CD77 was strongly expressed in HPC, HK2, and HRGECs, but not THP-1 monocytes, whereas TLR4 was expressed in THP-1 cells but with relatively weak levels in other cells (Fig. 3c, right). Taken together, these results suggest that, instead of CD77, TLR4 may serve as a receptor for mediating monocyte–Stx2 binding. Varrone et al. [7] discovered that the cleavage state of the A subunit affects the binding of Stx2 to monocytes and TLR4. Subsequently, we performed protein docking involving TLR4 and Stx2-B. Specifically, we obtained the extracellular protein sequence of TLR4 (O00206) and the protein sequence of Stx2-B (P09386) from the Swiss-Prot database. Employing the AlphaFold server, we established a protein docking model. This protein docking structure, visualized using the PyMOL software, unveils notable binding interactions between TLR4 and Stx2-B (Supplementary Fig. S4). To validate this, we tested whether the binding of Stx2-B-FITC with monocytes can be blocked by an inhibitory anti-TLR4 antibody. As shown in Fig. 3d, TLR4 blockade resulted in a significantly lower association of Stx2-B-FITC with THP-1 monocytes.

### EDL933 infection induces the infiltration of Stx2-positive monocytes in mouse kidney

**Fig. 4:**
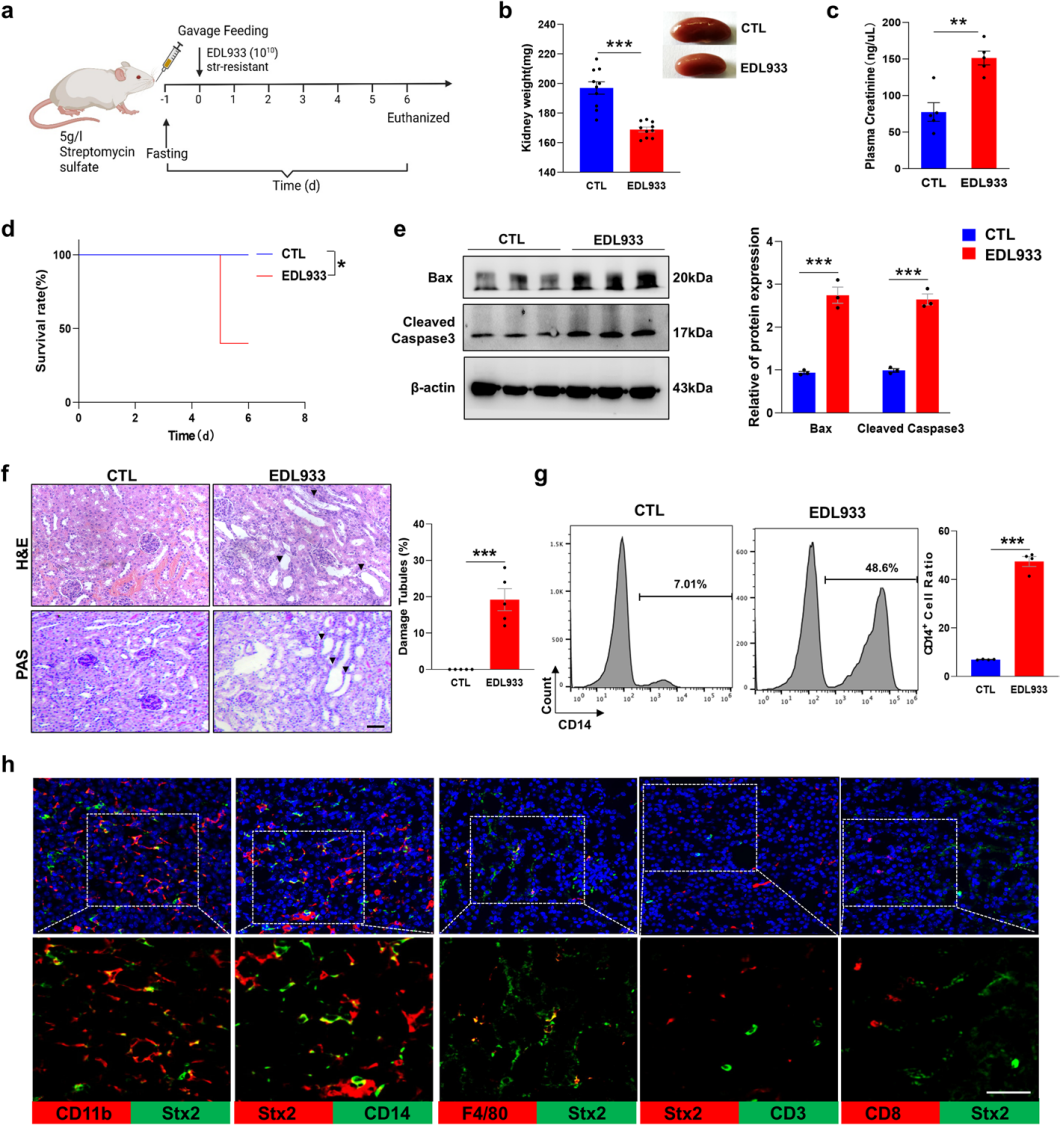
EDL933 infection activates monocytes and induces the infiltration of Stx2^+^ monocytes in mouse kidney. **a** The schematic of the experimental procedure. **b** Mouse kidney weight. **c** Mouse plasma creatinine level. **d** Survival rate. (*n* = 5 for mice in each group). **e** Levels of pro-apoptotic proteins (Bax and cleaved Caspase-3) in mouse kidney tissues. **f** Left; kidney injury detected by PAS and H&E staining, scale bar, 50 μm. Right; quantification of histopathological analysis. **g** Levels of CD14-positive monocytes in mouse peripheral blood. **h** Renal infiltration of Stx2-positive monocytes (but no other immune cells) detected by immunofluorescence label. In each group, there were 8–10 mice examined. The DAPI staining highlights cell nuclei (blue). Scale bar, 20 μm. Statistical differences between groups in panel **b**, **f** and **g** were assessed by two-tailed unpaired *t* tests. Statistical differences between groups in panel **d** were assessed by log-rank (Mantel–Cox) tests. Statistical differences between groups in panel e were assessed by two-way ANOVA (followed by Bonferroni’s multiple comparisons test). Data are presented as mean ± SEM. *P < 0.05; **P < 0.01; ***P < 0.001

To explore the potential role of monocytes in delivering Stx in HUS development after STEC infection, a murine HUS model was used [30]. In this experiment, EDL933, a STEC strain expressing both Stx1 and Stx2 [31], was used to infect male CD1 mice through gavage feeding (Fig. 4a). As shown in Fig. 4b, significant loss of kidney weight was observed in EDL933-infected mice. Supporting the observation of kidney injury induced by STEC infection, plasma creatinine levels in EDL933-infected mice were markedly increased (Fig. 4c). EDL933 infection also caused mouse death (Fig. 4d). Western blot analysis further showed that compared with non-infected mouse kidney, EDL933-infected mouse kidney had higher pro-apoptotic proteins, Bax and cleaved Caspase-3, suggesting a higher number of apoptotic cells and greater damage in EDL933-infected mouse kidney (Fig. 4e). The EDL933 infection-induced mouse kidney damage was further confirmed by PAS and H&E staining (Fig. 4f).

Along with the kidney injury, we also detected significantly higher levels of CD14^+^ monocytes in peripheral blood in EDL933-infected mice than in control mice (Fig. 4g). Activation of monocytes during the STEC infection is in agreement with previous reports [32, 33]. Moreover, co-localization of Stx2 and CD14 or CD11b was widely distributed in the kidney of EDL933-infected mice. However, co-localization of Stx2 and F4/80 (macrophages), CD3 (T cells) or CD8 (CD8 T cells) was scarcely detected (Fig. 4h).

### Depletion of monocytes attenuates LPS/Stx2-induced mouse HUS

**Fig. 5:**
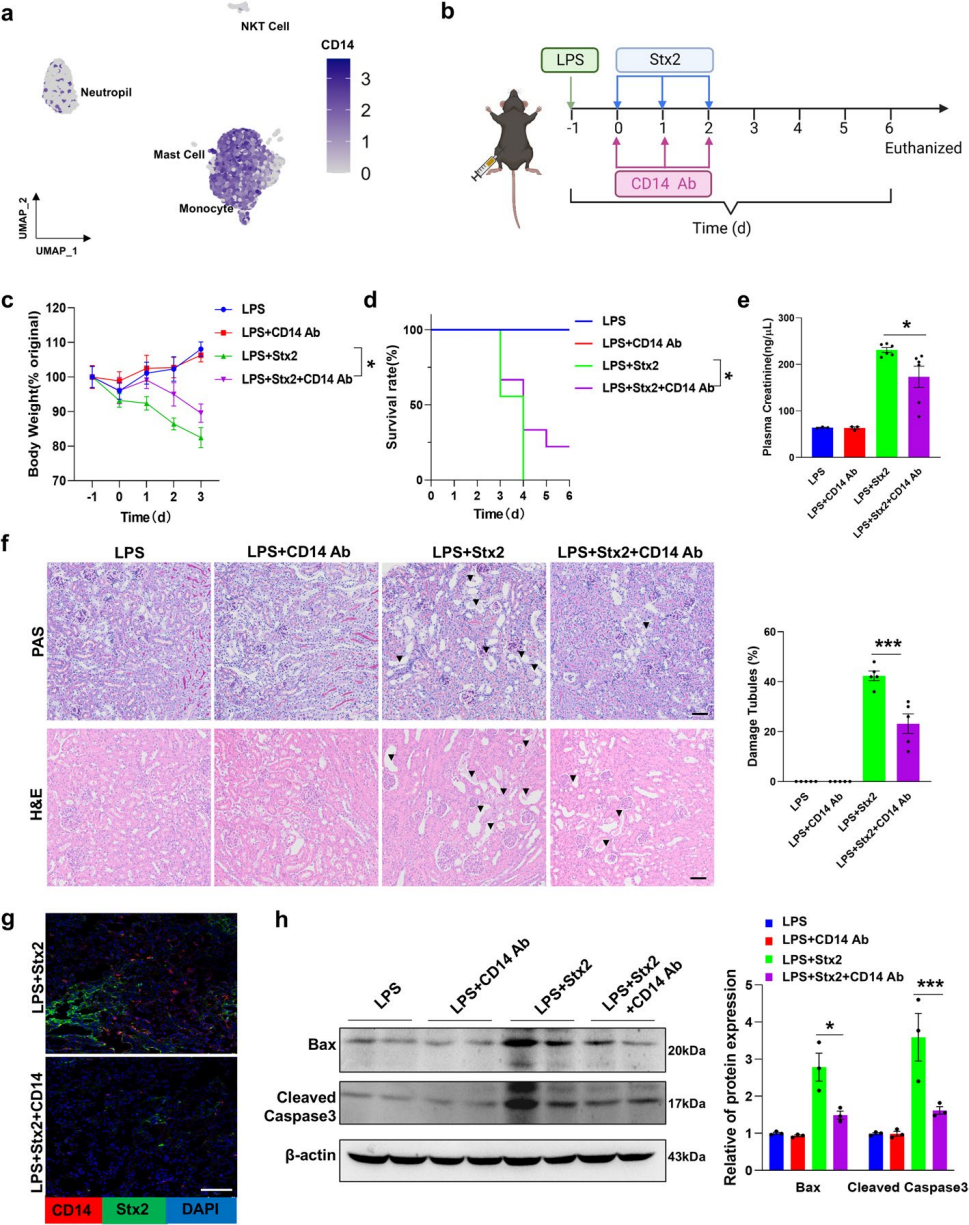
Therapeutic effect of CD14 neutralizing antibody on the LPS/Stx2-induced mouse HUS model. **a** The CD14 gene as seen in UMAP. **b** The experimental scheme. **c**, **d** Mouse weight loss (**c**) and survival rate (**d**) throughout HUS induction and the therapeutic process (LPS, *n* = 6; LPS + CD14, *n* = 6; LPS + Stx2, *n* = 9; LPS + Stx2 + CD14, *n* = 9). **e** Mouse plasma creatinine. **f** Left; PAS (upper) and H&E (lower) staining of mouse kidney tissue sections. Scale bar, 50 μm. The arrowheads indicated the tubular injury. Right; quantification of histopathological analysis. **g** Immunofluorescence co-staining of Stx2 with F4/80 in renal sections from mice with HUS induction and the therapeutic process. Scale bar, 20 μm. **h** Levels of pro-apoptotic proteins (Bax and cleaved Caspase-3) in mouse kidney. Statistical differences between groups in panel **e** and **f** were assessed by one-way ANOVA (Tukey’s multiple comparisons test). Statistical differences between groups in panel **c** were assessed by log-rank (Mantel–Cox) test. Statistical differences between groups in panel **h** were assessed by two-way ANOVA (followed by Bonferroni’s multiple comparisons test). Data are presented as mean ± SEM. *P < 0.05. ***P < 0.01

Single-cell gene sequencing analysis of the Stx2-B-positive cells confirmed that monocytes have the highest levels of CD14 expression (Fig. 5a). Given the prior finding that Stx2 is delivered by monocytes, we hypothesized that depleting monocytes by anti-CD14 neutralization antibody might block the transport of Stx2 to the kidney and thus attenuate the renal damage in the LPS/Stx2-induced murine HUS model (Fig. 5b) [17]. In agreement with previous reports [17], LPS/Stx2 treatment strongly reduced mouse body weight. However, CD14 neutralizing antibody displayed a significant effect in slowing down the LPS/Stx2-induced bodyweight loss (Fig. 5c). The mortality of mice caused by LPS/Stx2 treatment was also markedly reduced by the CD14 neutralizing antibody (Fig. 5d).

Mouse renal function assays were performed by measuring creatinine levels in plasma. As shown, LPS/Stx2 treatment strongly increased plasma creatinine levels (Fig. 5e), whereas such increased creatinine levels by LPS/Stx2 was reduced by CD14 neutralizing antibody treatment. The protective effect of CD14 neutralizing antibody treatment on LPS/Stx2-induced mouse kidney damage was validated by PAS (Fig. 5f, top) and H&E (Fig. 5f, bottom) staining. Co-staining Stx2 with CD14 in kidney tissue sections from the mice subjected to different treatments revealed the presence of numerous infiltrated Stx2-positive monocytes in the mouse kidney; however, such renal infiltration of monocytes and Stx2 was largely abolished by CD14 neutralizing antibody treatment (Fig. 5g). We also found that renal Bax and cleaved Caspase-3 levels were markedly increased by LPS/Stx2 treatment (Fig. 5h), suggesting LPS/Stx2-induced renal cell apoptosis. However, CD14 neutralizing antibody treatment almost completely abolished the induction of Bax and cleaved Caspase-3 by LPS/Stx2 treatment (Fig. 5h).

### Blockade of monocyte renal infiltration mitigates LPS/Stx2-induced mouse HUS

**Fig. 6:**
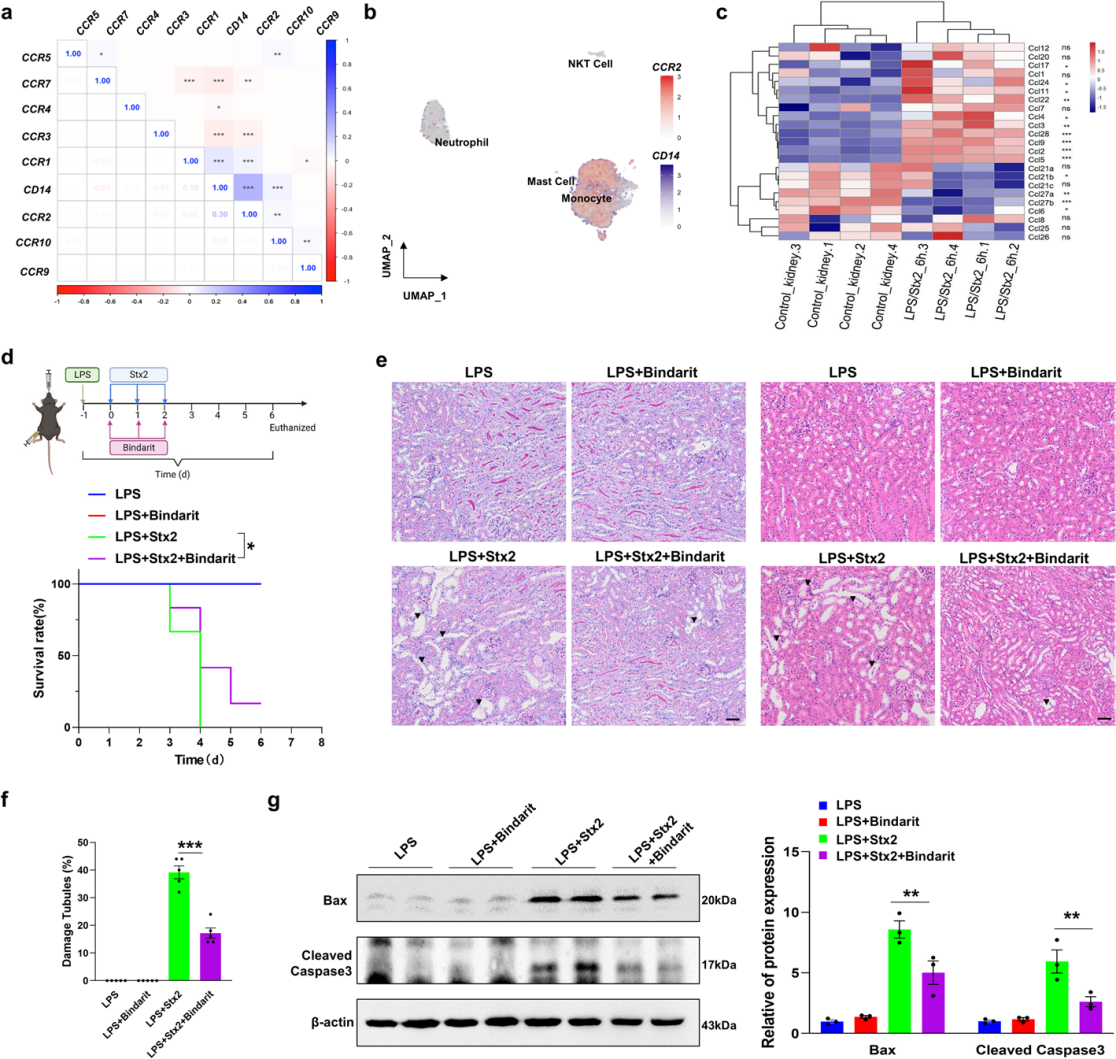
Therapeutic effect of bindarit on the LPS/Stx2-induced mouse HUS model. **a** The correlation of *CD14* and *CCRs* in Stx2^+^ cells. **b** *CD14* and *CCR2* expression in Stx2^+^ cells. **c** Heatmap of *Ccl* family gene expression in renal tissues treated with control or LPS/Stx2 for 6 h. **d** The experimental scheme (upper) and mice survival rate throughout the HUS induction and therapeutic process (lower, LPS, *n* = 5; LPS + bindarit, *n* = 5; LPS + Stx2, *n* = 12; LPS + Stx2 + bindarit, *n* = 12). **e** Renal sections from mice were subjected to PAS (left) and H&E (right) staining. Scale bar, 50 μm. The arrowheads indicate the tubular injury. **f** Quantification of histopathological analysis. **g** Levels of pro-apoptotic proteins (Bax and cleaved Caspase-3)in renal tissue during HUS induction and the therapeutic process with statistical analysis. Statistical differences between groups in panel **d** were assessed by log-rank (Mantel-Cox) test. Statistical differences between groups in panel **f** were assessed by one-way ANOVA (Tukey’s multiple comparisons test). Statistical differences between groups in panel **g** were assessed by two-way ANOVA (followed by Bonferroni’s multiple comparisons test). Data are presented as mean ± SEM. *P < 0.05. **P < 0.01. ***P < 0.001

It is well-known that CCR2 serves as a chemokine receptor guiding monocyte chemotaxis [34]. We analyzed the correlation between CD14 and various CCRs in Stx2-positive cells isolated from peripheral blood to explore whether CCR2 is involved in the monocyte infiltration during Stx2 infection. As shown, CCR2 and CD14 had a high degree of overlap, suggesting expression of high level of CCR2 in monocytes (Fig. 6a, b). In contrast, other CCR receptors did not exhibit a significant overlap with CD14 (Supplementary Fig. S6).

We further analyzed the RNAseq data (GSE180476) from the mouse HUS model study (6 h post-treatment with LPS/Stx2) conducted by Kume et al. [23] to explore the chemoattractant involved in monocyte renal infiltration. The heatmap of Ccl family gene expression in renal tissues treated with or without LPS/Stx2 revealed that levels of *Ccl2*, as well as other chemokines *Ccl3*, *Ccl4*, and *Ccl5*, etc., were increased at 6 h post-LPS/Stx2 treatment (Fig. 6c). As Stx2-positive monocytes express high levels of CCR2, they can infiltrate into renal tissues that generate large amounts of chemokine CCL2 (monocyte chemotactic protein 1, MCP-1) [23]. We thus postulated that inhibiting the CCR2/CCL2 signal pathway should block the monocyte renal infiltration, leading to mitigation of LPS/Stx2-induced mouse kidney injures. To test this hypothesis, we reduced CCL2 levels and consequent monocyte renal infiltration using bindarit, a small molecule that inhibits CCL2 synthesis [35]. We administered mice with bindarit (100 mg/kg/day) via gavage each day for 3 days after LPS injection (Fig. 6d, top). As shown in Fig. 6d, bottom, treatment with bindarit significantly increased the survival rate of mice treated with LPS/Stx2. This result is in agreement with the previous finding that MCP-1 may play a critical role in the pathogenesis of HUS through the recruitment and activation of monocytes [36].

The therapeutic effects of bindarit on LPS/Stx2-induced mouse HUS was further analyzed by PAS (Fig. 6e, left) and H&E (Fig. 6e, right) staining of mouse renal tissue sections, and demonstrated that bindarit protects against kidney injures (Fig. 6f). Supporting this, western blot analysis of the level of apoptotic proteins Bax and cleaved Caspase-3 in kidney tissue of LPS/Stx2-treated mice showed that bindarit effectively reduced renal cell apoptosis (Fig. 6g). These results collectively suggest that blockade of the synthesis of monocyte chemoattractant CCL2 by bindarit effectively prevents monocytes from transporting Stx2 to the kidney and causing kidney damage.

## Discussion

STEC are noninvasive bacteria restricted to the intestines. To cause renal damage, STEC-produced Stx must be transported from the intestines and guts to the kidneys. Several studies have reported that HUS patients had higher free Stx2 than non-HUS patients, suggesting a possible involvement of high levels of free Stx2 in HUS development [5–8]. It is likely that cell-free toxin executes its function differently from the cell-delivery toxin in STEC infection-induced HUS development, and further study is required to establish the direct correlation between the free toxin in the bloodstream and the onset of HUS. Employing mouse HUS models induced by LPS/Stx2 or directly by EDL933 infection, we screened various peripheral leukocytes for delivering Shiga toxin from infected intestine to kidney during HUS development. Single-cell sequencing and cell co-incubation assays both identified peripheral monocytes as major carriers of Stx in STEC infection-induced HUS development. The critical role of monocytes in facilitating HUS was further validated by an intervening strategy in which depleting peripheral monocytes via CD14 neutralizing antibody, or blocking monocyte renal infiltration by CCL2 (MCP-1) inhibition, significantly attenuated kidney injury and dysfunction induced by LPS/Stx2 treatment.

Identification of circulating monocytes as potential Stx carriers in STEC-induced HUS disease is in agreement with previous studies by different investigators [37, 38]. As an important innate immune cell population, monocyte inflammatory responses are a major component of STEC infection-induced severe enteritis [39]. A study by Brigotti et al. illustrated a critical role of circulating monocytes in the very early phases of the pathogenic process culminating in HUS by releasing large amounts of proinflammatory molecules [32]. Additionally, Pohl et al. demonstrated that CCR2-dependent Gr1^high^ monocytes promote kidney injury in STEC-induced HUS in mouse models [40]. Several pieces of evidences in the present study support the notion that circulating monocytes, but not populated neutrophils, deliver Stx from infected intestine to kidney: Firstly, comparison of the binding affinity of Stx2-B to human peripheral monocytes and neutrophils showed that monocytes possess significantly higher affinity to Stx2-B than neutrophils, which is in line with the conclusion drawn from single-cell sequencing analysis; secondly, cell binding assays showed that Stx2-B-bearing human peripheral monocytes or THP-1 monocytes could pass Stx2 to renal endothelial HRGECs, and induced HRGEC apoptosis; thirdly, a significant infiltration of Stx2-bearing monocytes in the renal tissues of mice treated with EDL933 strain was observed. This is in line with the single-cell analysis that monocytes express high level of CCR2, and the mouse kidney tissues secrete large amounts of CCL2 (MCP-1) following LPS/Stx treatment. Through interacting with its receptor CCR2, CCL2 guides monocyte infiltration into kidney tissues; finally, the intervention strategies further validated the role of monocytes transferring the toxin from the infected intestine to kidney in mice treated with LPS/Stx. Supporting this, depleting circulating monocytes with CD14 neutralizing antibody significantly mitigated mouse kidney injures induced by LPS/Stx2 treatment. In addition, suppression of CCL2 level by bindarit also attenuated the LPS/Stx2-induced mouse kidney injures via blocking monocyte recruitment and infiltration in the kidney.

Recent investigations have revealed the presence of Stx in circulating leukocytes and their secreted extracellular vesicles (EVs) [5, 19]. In a study examining sera from children with STEC infection, Brigotti et al. [5] identified the association of Stx with circulating neutrophils and their released EVs. Ståhl et al.[19] also demonstrated the involvement of leukocyte-derived EVs in bacterial toxin transfer, showing that these Stx-laden EVs undergo endocytosis in glomerular endothelial cells, leading to cell death. Contrary to these findings, our cell–toxin binding experiments using human monocytes, neutrophils, and THP-1 monocytes revealed that toxins predominantly remained on the cell surface following incubation with Stx2-B-FITC. Only a small amount of Stx2-B-FITC was detected on the EVs released by peripheral monocytes or THP-1 monocytes after a 6-h treatment with the toxin. Notably, the EVs released by Stx2-B-bearing monocytes failed to induce significant apoptosis of HRGECs. Our results suggest that monocyte-released EVs may not play a major role in the initial transfer of toxins. However, it is possible that Stx-bearing monocytes may release Stx-positive EVs after infiltrating kidney tissues. Further investigations are needed to explore this aspect comprehensively.

Previous studies have shown that circulating leukocytes primed by LPS or under inflammatory condition possess higher affinity to Shiga toxin [17, 41, 42]. Clayton and co-workers showed that LPS upregulated Stx receptors in a primate model of HUS [43]. It is well known that, in addition to Stx administration, prior systemic LPS administration allows for more reliable induction of HUS that mimics pathological manifestations in human disease [44, 45]. LPS priming is certainly consistent with the protocol for inducing HUS in mice, which entails peritoneal exposure to both LPS and Stx2 [25, 44–46]. In contrast, the mere administration of Stx2 induces partial symptoms of HUS which are also less severe. In line with this, we found that the number of Stx2-bearing monocytes was strikingly increased in both peripheral blood and kidney in Stx2/LPS-treated mice, suggesting that LPS priming may facilitate HUS development via increasing the binding affinity of monocytes to Stx2 or the chemotaxis of monocytes to mouse kidney.

Given the observed disparity in the affinity of monocytes and renal endothelial HRGECs to Stx2, it is evident that distinct receptors facilitate the association of Stx2 with these cell types. Notably, the lower affinity of monocyte–Stx binding compared with HRGEC–Stx binding implies the potential transfer of Stx from monocytes to HRGECs. While the role of Gb-3/CD77 as a receptor for Stx binding to renal endothelial cells is well-accepted, the identity of the receptor mediating monocyte–Stx binding remains elusive. Supporting the concept that circulating monocytes function as carriers of Stx, previous studies have shown that circulating monocytes express globotriaosylceramide (Gb3Cer) or a Gb3 species that is different from that found on endothelial cells, probably a short-chain fatty acyl Gb3 [37]. Brigotti et al. [32] demonstrated that human monocytes, when stimulated by Stx1a via the glycolipid receptor globotriaosylceramide, release various pro-inflammatory cytokines associated with HUS. In comparison with Stx1a-treated neutrophils, primary human monocytes stimulated with Stx1a released significantly higher levels of pro-inflammatory cytokines, including IL-1β, TNFα, IL-6, G-CSF, CXCL8, CCL2, and CCL4, consistent with the RNAseq results. However, our investigation did not reveal high levels of CD77 (Gb3) expression in monocytes/macrophages in either kidney tissue or peripheral blood. Instead, our results suggest that TLR4, which is prominently expressed in monocytes, may act as a receptor for Stx2. Notably, the binding of Stx2-FITC to monocytes was strongly inhibited by an anti-TLR4 antibody.

**Fig. 7:**
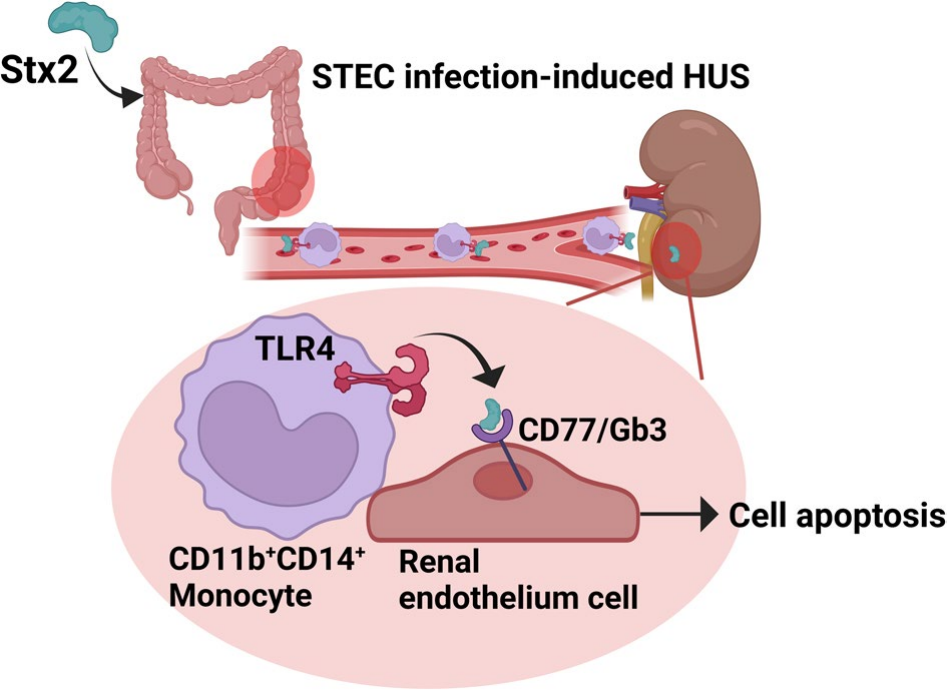
Schematic working model of monocytes delivering Stx2 from the infected intestines to the kidney during hemolytic uremic syndrome development

In summary, our data support a working model of Stx-induced hemolytic uremic syndrome depicted in Fig. 7. Under Shiga toxin-producing *E. coli* infection, monocytes may initially bind to Stx2 through TLR4 in the infected intestinal region. Subsequently, they may circulate through the bloodstream, infiltrate the kidney, which secretes chemoattractant for recruiting monocytes. Within kidney tissues, monocytes may transfer Stx2 to renal endothelial or tubular cells, inducing renal cell apoptosis and tissue injuries.

## Supplementary Information

Supplementary Material 1.

## Data Availability

Single-cell transcriptomic data supporting the findings of this study are openly available in GSE 252352. The code used in the analyses is available with application in https://github.com/Xinlei672/Stx2-cell.git. Uncropped western blot images and statistical source data are provided as supplementary files. Further supporting data are available upon request to the corresponding authors Ke Zen (kzen@nju.edu.cn).

## Abbreviations

Stx: Shiga toxin
HUS: Hemolytic uremic syndrome
STEC: Shiga toxin (Stx)-producing *Escherichia coli*
A4GALT: Alpha 1,4-galactosyltransferase
TLR4: Toll-like receptor 4
HRGEC: Human renal glomerular endothelial cells
HPC: Human podocyte cell line
