# Supplementary material for: Monocytes serve as Shiga toxin carriers during the development of hemolytic uremic syndrome
Source: Cell Mol Biol Lett. 2025 Jan 27;30:13. doi: 10.1186/s11658-025-00689-8 (PMC11773931; doi:10.1186/s11658-025-00689-8)
Supplement: Supplementary file 1 — Supplementary Material 1. [file 11658_2025_689_MOESM1_ESM.docx]

Supplementary file for

**Monocytes serve as Shiga toxin carrier during the development of hemolytic uremic syndrome**

Xinlei Sun^1*ᵠ^, Shuang Qu^2*^, Fenglian Zhou^1^, Fujie Shi^1^, Yunfei Wu^3^, Lin Gu^4^, Minghui Liu^3^, Zhen Bian^1^, Lei Shi^1^, Zhihong Liu^5¶^, Yuan Liu^1¶^ and Ke Zen^1,6¶^

**File list**

1. Supplementary Figure S1
2. Supplementary Figure S2
3. Supplementary Figure S3
4. Supplementary Figure S4
5. Supplementary Figure S5
6. Supplementary Figure S6


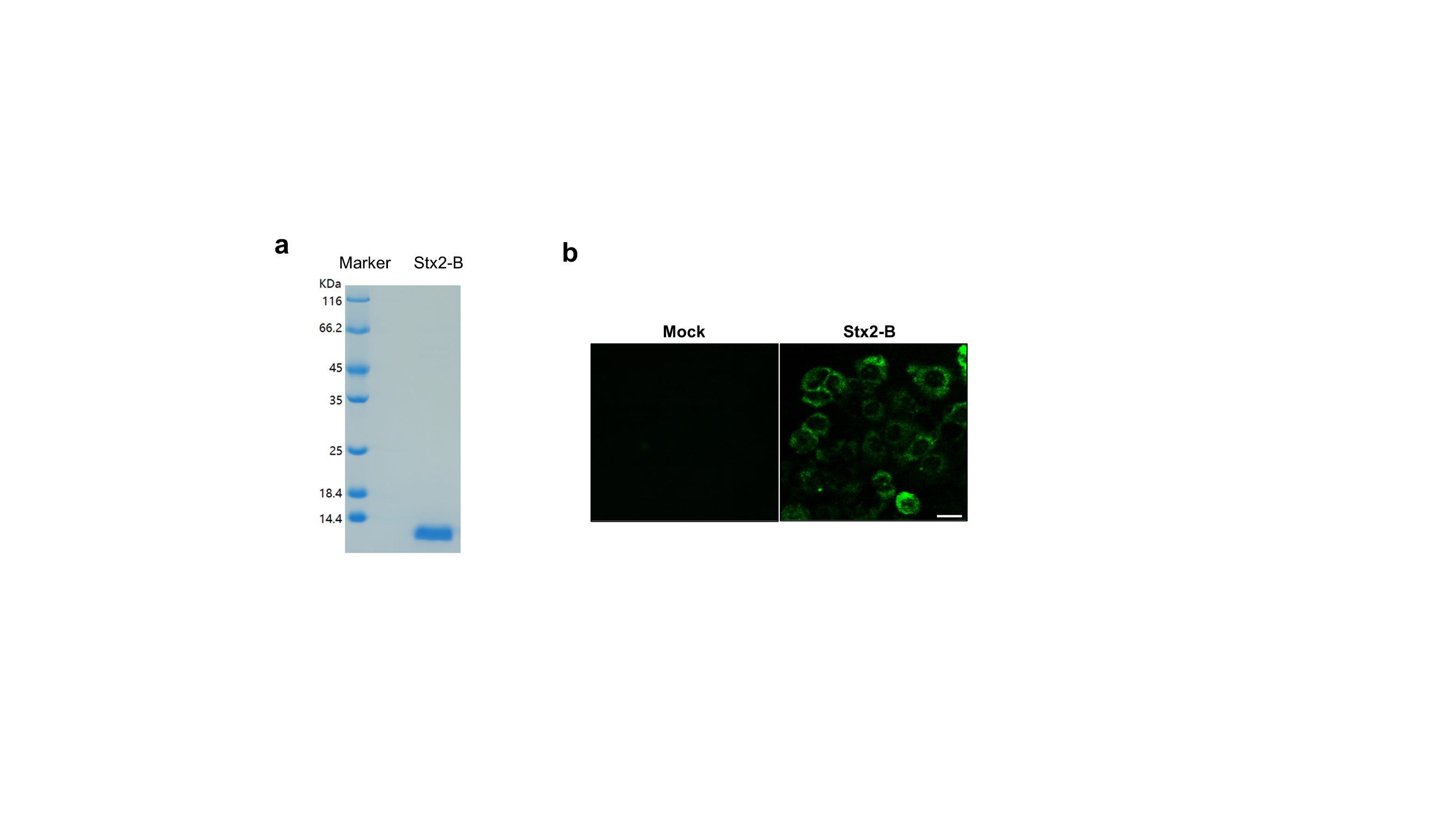


**Supplementary Figure S1. Stx2-B-FITC production and functional verification. a,** Purification of Stx2-B protein from the EDL933 strain, with gel staining using Coomassie blue. **b,** Stx2-B-positive HeLa cells detected by immunofluorescence after incubating cells with Stx2-B FITC (100ng/ml) for 30 min. Scale bar, 100μm.


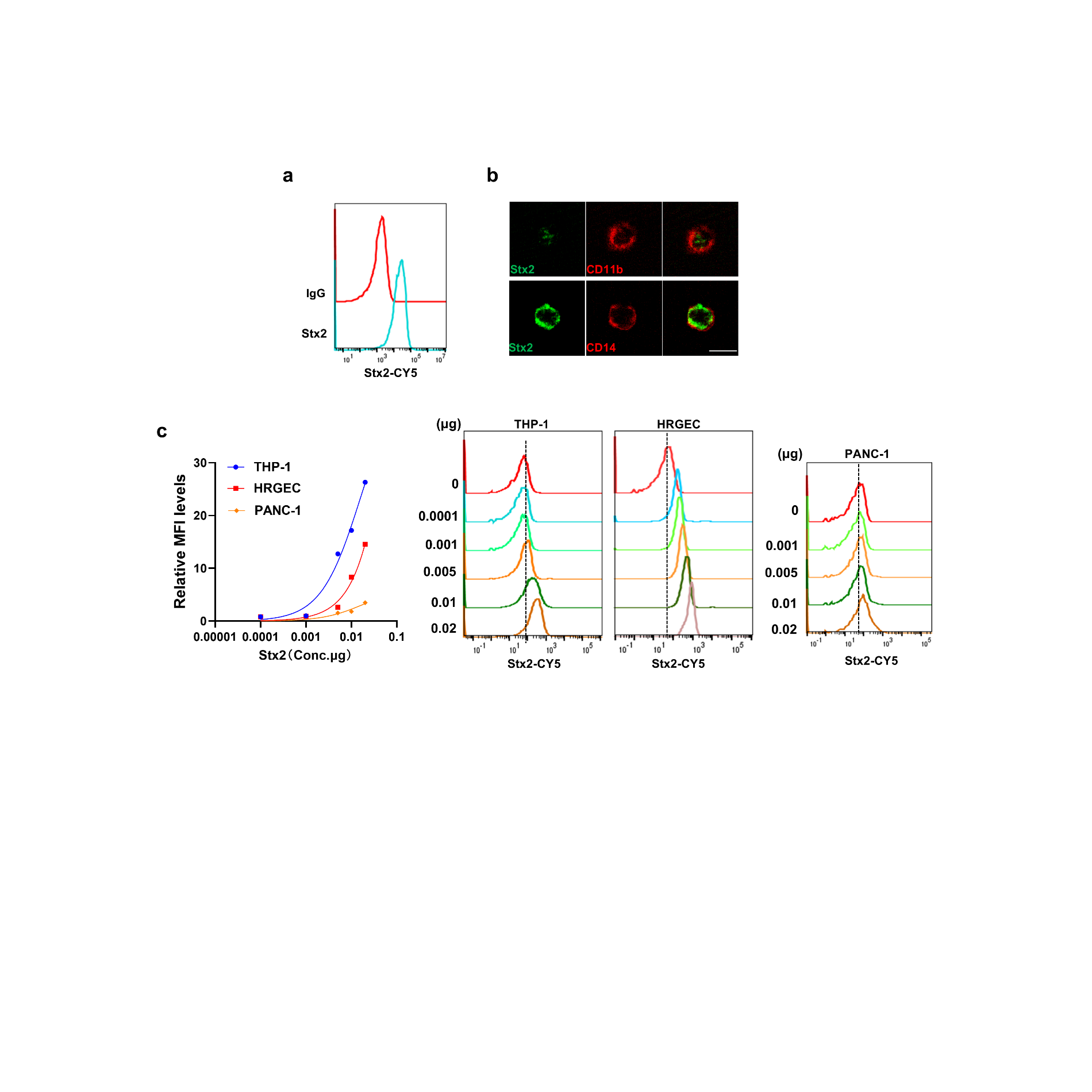


**Supplementary Figure S2. Stx2-Cy5 binding affinity to THP-1 and HERBC cells. a,** Stx2 protein was conjugated with CY5, and the binding efficiency of Stx2-CY5 to HeLa cells was validated through flow cytometry assays by incubating cells with Stx2-B FITC (2 ng/ml) for 30 min. **b,** Stx2-CY5 exhibited strong binding to CD14-positive cells. Scale bar, 10μm. **c,** Assessment of the binding capacity of Stx2 to THP-1 monocytes and HRGEC cells. PANC-1 (pancreatic cancer cells with low TLR4 and Gb3 expression) served as a negative control.

**
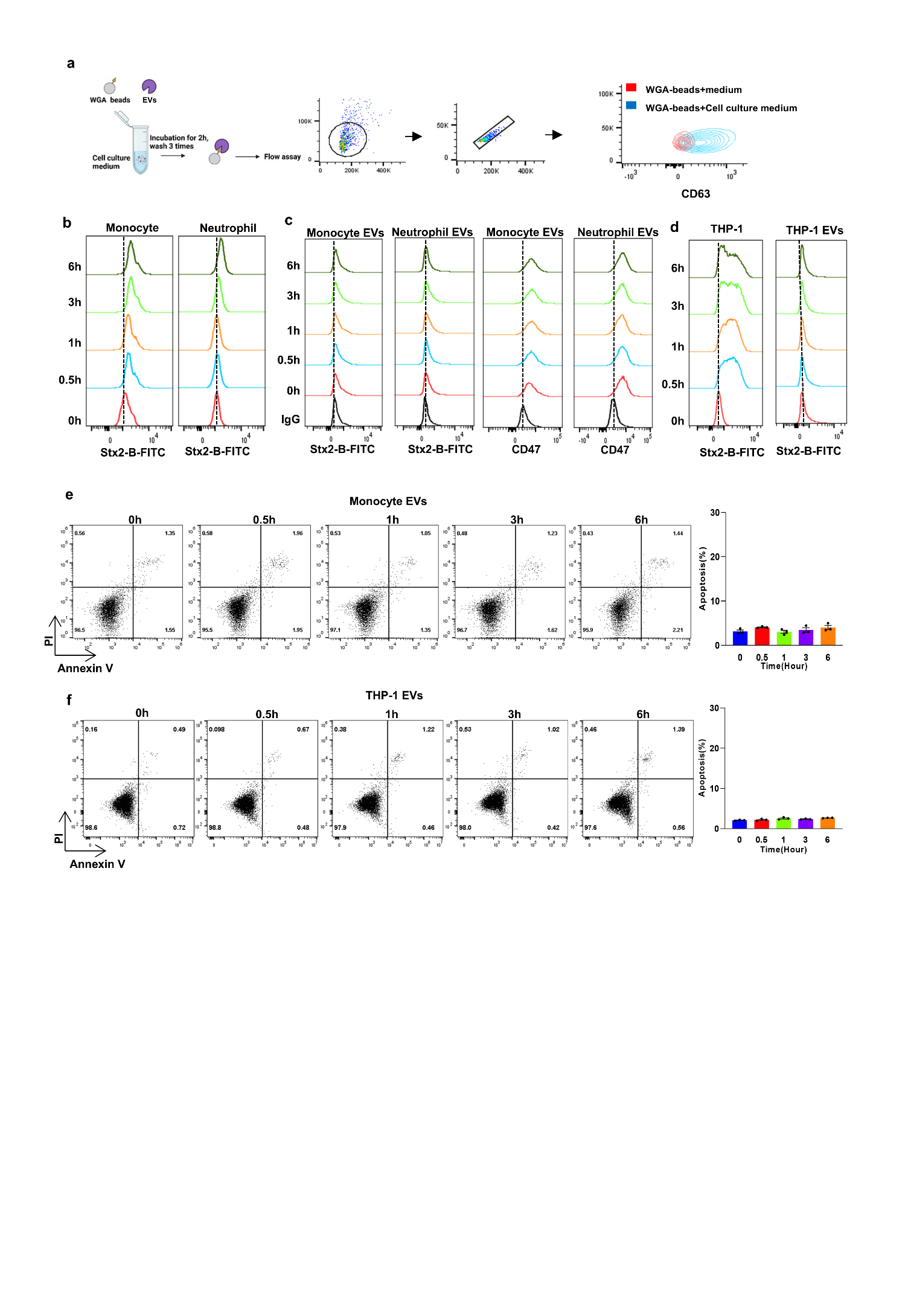
**

**Supplementary Figure S3. Monocyte and neutrophil extracellular vesicles are not contributed to carrying Stx2 to HRGEC cells. a,** The schematic of the experimental procedure. **b,** Detection of monocyte and neutrophil binding to Stx2-B at different time points. **c,** Detection of extracellular vesicle release from monocytes and neutrophils binding to Stx2-B at different time points (left). CD47 detection in extracellular vesicles of neutrophils and monocytes serving as a positive control (right). **d,** Evaluation of the binding ability of THP-1 cells (left) and release of vesicles by THP-1 cells when interacting with Stx2-B at different time points (right). **e,** Assessing the effect of extracellular vesicles released by Stx2-treated monocytes on HRGEC cells. **f,** Analyzing the impact of extracellular vesicles released by Stx2-treated THP-1 cells on HRGEC cells.


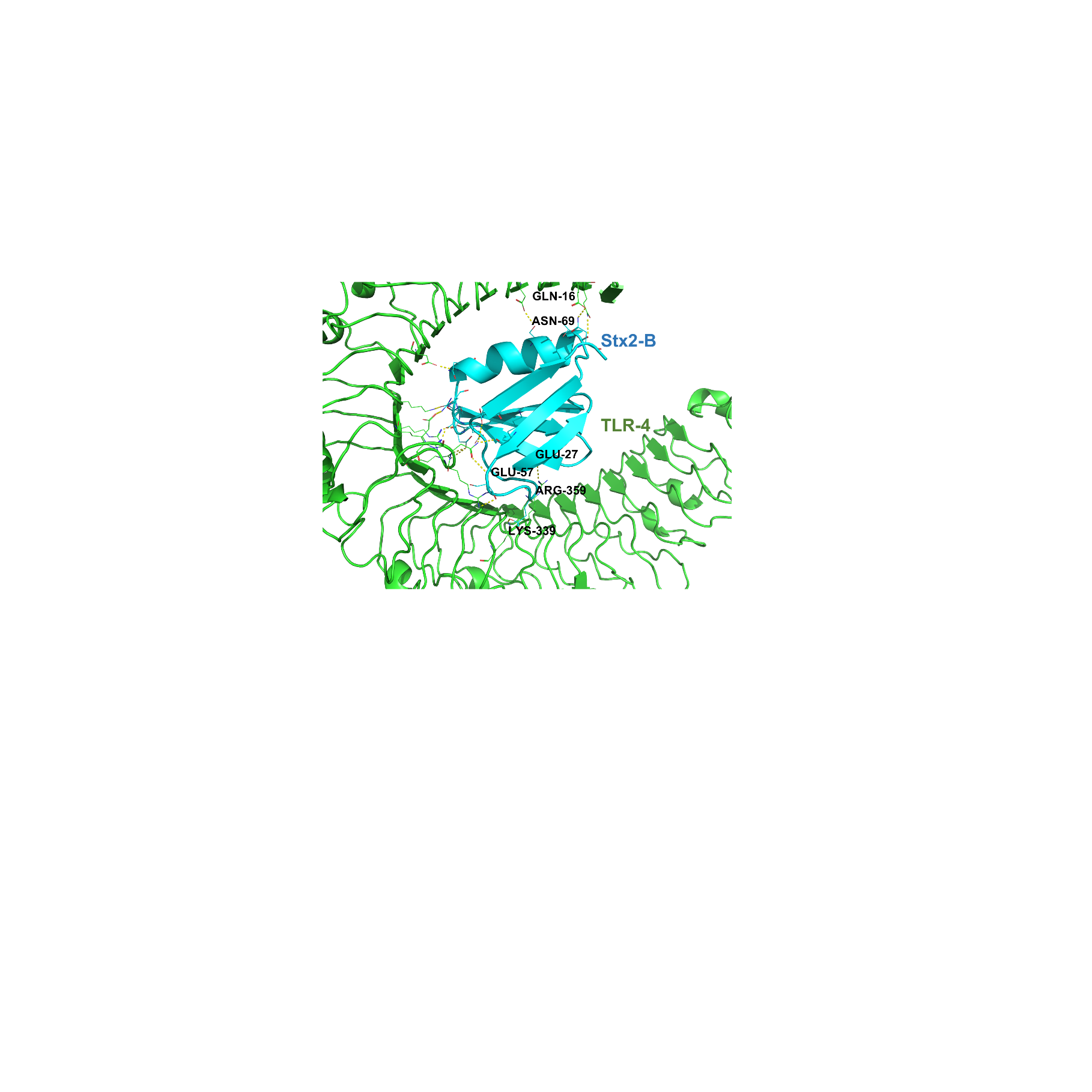


**Supplementary Figure S4.** **Docking of TLR4 with Stx2-B.** The extracellular protein sequence of TLR4 (O00206) and the protein sequence of Stx2-B (P09386) were retrieved from the Swiss-Prot database. The binding interactions between these two molecules were predicted using the AlphaFold Server and visualized using PyMOL software.


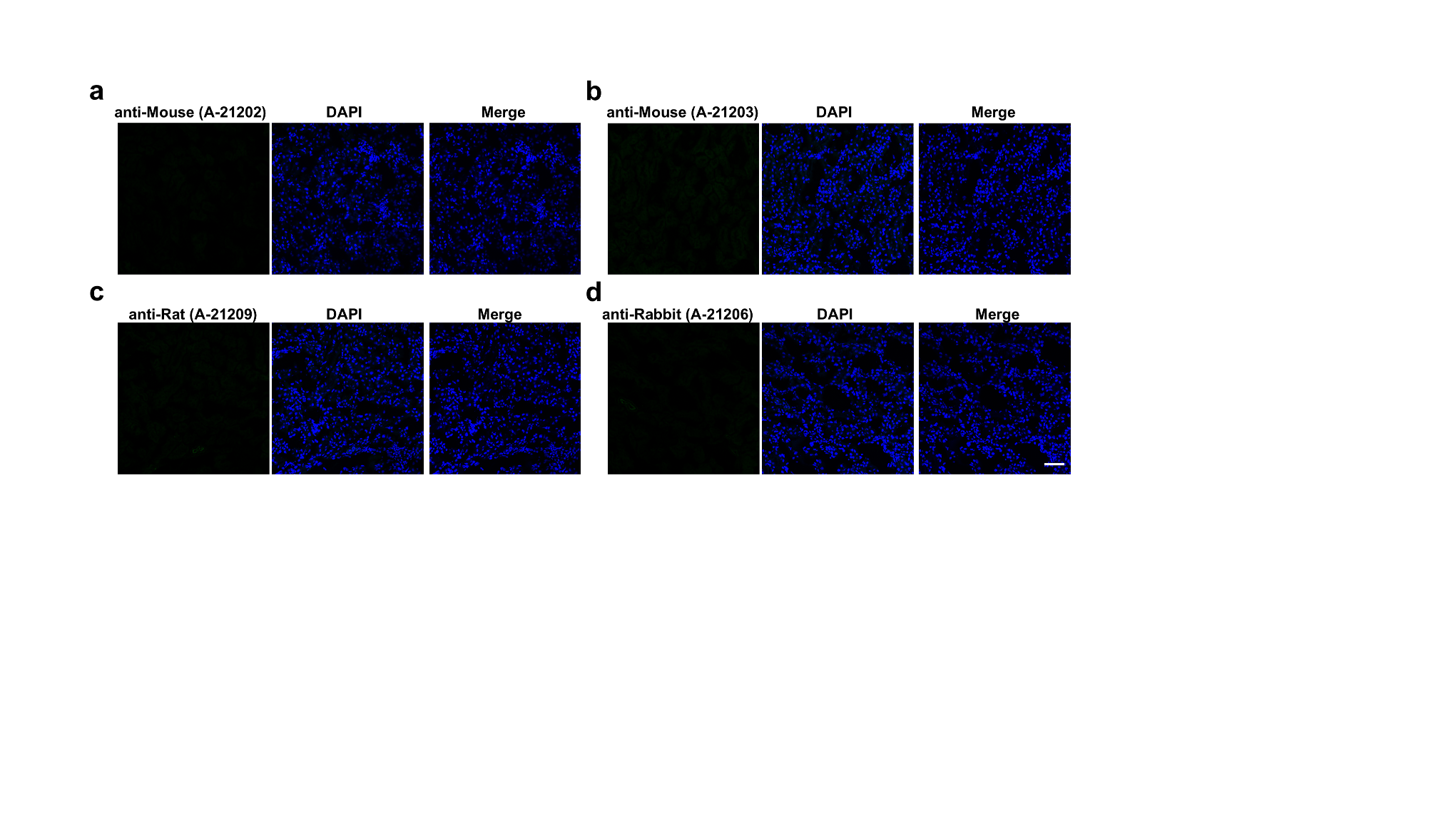


**Supplementary Figure S5. The kidney sections incubated with secondary antibody. a-d,** The tissue sections were obtained from EDL933 infection mouse kidney, treated with fluorescently labeled secondary antibodies, including anti-Mouse-Alexa Fluor™ 488 (a), anti-Mouse-Alexa Fluor™ 594 (b), anti-Rat-Alexa Fluor™ 594 (c) and anti-Rabbit-Alexa Fluor™ 488 (d). The DAPI staining highlights cell nuclei(blue). Scale bar, 50μm.


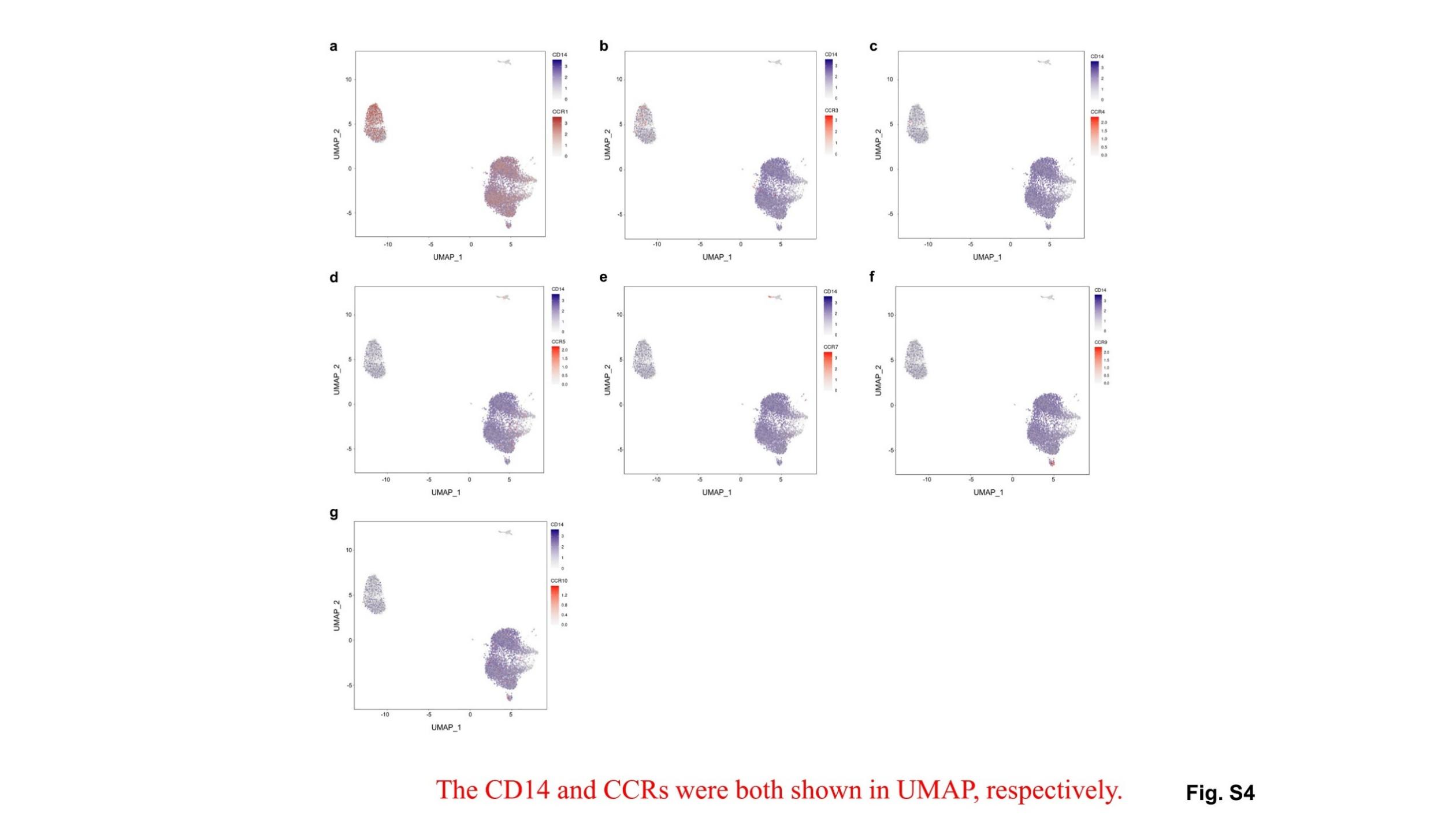


**Supplementary Figure S6. The CD14 and CCRs were both shown in UMAP, respectively. a-g,** The CD14 and CCR1(a), CCR3(b), CCR4(c), CCR5(d), CCR7(e), CCR9(f) and CCR10(g) expression were shown in UMAP, respectively.
